# Estimation of health utility values for alopecia areata

**DOI:** 10.1007/s11136-024-03645-9

**Published:** 2024-03-29

**Authors:** Daniel Aggio, Caleb Dixon, Ernest H. Law, Rowena Randall, Thomas Price, Andrew Lloyd

**Affiliations:** 1 Acaster Lloyd Consulting Ltd, 8th Floor, Lacon House, 84 Theobalds Road, London, WC1X 8NL UK; 2 Pfizer Inc, New York, NY USA; 3 Pfizer Ltd, Tadworth, UK

**Keywords:** Alopecia areata, Time trade off, Health state utility values, Health-related quality of life

## Abstract

**Purpose:**

Alopecia areata (AA) is an autoimmune-mediated inflammatory dermatological disease characterised by non-scarring hair loss affecting the scalp and sometimes other hair-bearing sites. This study aimed to elicit health state utility values (HSUVs) from the UK general population for AA using time trade off (TTO) interviews.

**Methods:**

Vignette descriptions of health states defined by the extent of hair loss were developed (as well as one describing caregiver burden). These were developed using data from standardised patient reported outcome (PRO) measures, a literature review and qualitative interviews. Health states were defined based on the severity of alopecia tool (SALT), which assesses extensiveness of scalp hair loss. HSUVs were then elicited for each health state in TTO interviews with the UK public.

**Results:**

One caregiver and five patient health states were developed based on the literature review findings, clinical trial PRO (Hospital Anxiety and Depression Scale and Alopecia Areata Patient Priority Outcomes Questionnaire) data and qualitative interviews with patients (*N* = 11), clinical experts (*N* = 4) and caregivers of adolescents with AA (*N* = 10). These data showed a more severe impact among patients with more extensive hair loss. One hundred and twenty participants evaluated the vignettes in TTO interviews. Patient HSUVs ranged from 0.502 for the most extensive hair loss health state (SALT 50–100 + eyebrow and eyelash loss) to 0.919 (SALT 0–10) for the mildest health state. The caregiver HSUV was 0.882.

**Conclusion:**

Quantitative and qualitative data sources were used to develop and validate vignettes describing different AA health states. Patient and caregiver HSUVs demonstrate a large impact associated with AA, especially for states defined by more extensive hair loss.

**Supplementary Information:**

The online version contains supplementary material available at 10.1007/s11136-024-03645-9.

## Introduction

Alopecia areata (AA) is an autoimmune-mediated inflammatory dermatological disease characterised by non-scarring hair loss, frequently involving the scalp but sometimes other hair-bearing sites, including eyebrows, eyelashes, facial and body hair [1]. Scalp hair loss due to AA can range from well-defined patches to diffuse or total hair loss [1, 2]. The most common form is patchy AA, characterised by one or multiple patches of hair loss. AA can lead to total or near total scalp hair loss (alopecia totalis) and sometimes total or near total loss of hair on the whole body (alopecia universalis) [1]. The extent of hair loss can be estimated using the Severity of Alopecia Tool (SALT), a standardised measure assessed by a physician, grading extensiveness of scalp hair loss from 0 to 100, with higher scores reflecting more extensive hair loss [3]. AA is a prevalent disease with a 2019 estimate of 18.4 million cases worldwide [4] and a UK point prevalence of 0.58% in 2018 [5].

AA can impact a patient’s health-related quality of life (HRQoL), particularly in psychosocial domains [6–9]. AA can lead to social anxiety, self-consciousness and reduced participation in social and physical activities [10–13]. Patients with AA may also experience depression [6, 14]. Physical discomfort and pain have also been described due to skin irritation, itching and nail weakness [15, 16]. More extensive hair loss is also associated with more severe HRQoL impairment [6–9].

Few effective treatments exist for AA [17]. Corticosteroids and contact immunotherapy are recommended by the British Association of Dermatologists (BAD), but these therapies are being used off-licence and are therefore not suitable for long-term treatment [17]. The BAD acknowledge the need for an effective treatment to ameliorate the psychological effects of AA [17]. New treatments are in development that act on the physiological cause of the disease [18–21]. Decision makers, especially in health technology assessment (HTA) agencies, will assess the value of any treatment in AA. Part of that value will relate to the impact of treatments on HRQoL. Evidence demonstrating a link between the extent of hair loss and deterioration in HRQoL is important for understanding the potential benefits of treatment [6–9].

Where HTA bodies use cost-effectiveness analyses to understand the value of a treatment, preference weighted HRQoL data (or utility weights) are typically required. The EQ-5D is generally the preferred method for estimating utility weights and is advocated by several decision makers, including the National Institute for Health and Care Excellence (NICE, a UK-based HTA body) [22]. The EQ-5D is a generic measure of health, assessing mobility, self-care, usual activities, pain/discomfort and anxiety/depression. In a recent review, the NICE concluded that: “the EQ-5D often fails to capture quality-of-life improvements for people with skin conditions” [23]. Empirical evidence also suggests the EQ-5D may be insensitive to the full impact of AA [24, 25]. The NICE recommend that alternative methods for deriving utilities are used where the EQ-5D has been shown to be inappropriate [26]. One such method is the vignette approach, whereby researchers develop descriptions of how a condition impacts patients’ HRQoL. These descriptions are then valued using one of several valuation techniques (e.g. the time trade off [TTO] method).

Vignette-based methods have been used to estimate utilities used in economic analyses for conditions and treatment attributes that are difficult or impossible to obtain via preference-based instruments, mapping functions or published literature [27]. Published guidance recommends developing vignettes using multiple sources of high-quality evidence including publicly available citations, qualitative data and validated patient reported outcome (PRO) measures of HRQoL [26, 27]. Once drafted, vignettes should undergo multiple rounds of review with clinicians and patients to refine content and maximise accuracy [26–28].

This study aimed to develop and utilise AA health state vignettes to estimate UK public health state utility values fit-for-purpose in economic evaluations of AA treatments based on best practice recommendations.

## Methods

### Study design

This study was comprised of two parts. Part one developed vignette descriptions of patients with AA and caregiver health states using multiple sources of evidence (Table 1). Part two elicited the views of 120 members of the UK public regarding the severity of the vignettes using the TTO method. Five adult AA vignettes defined by SALT score and a caregiver vignette were developed. This study was approved by the Western Institutional Review Board on 17 May 2022 (study number: 1333147) prior to participant recruitment.

**Table 1:** Data sources for vignette development by health state

Adult patient and caregiver health states	Sources for vignette development
≥ 50% scalp hair loss (SALT 50–100) and eyebrow and eyelash hair loss^a^	• AAPPO/HADS • Literature review/study sponsor materials • Patient interview data
≥ 50% scalp hair loss (SALT 50–100)	• AAPPO/HADS • Literature review/study sponsor materials • Patient interview data
21–49% scalp hair loss (SALT 21–49)	• AAPPO/HADS • ≥ 50% hair loss vignette • Literature review/study sponsor materials • Patient interview data
11–20% scalp hair loss (SALT 11–20)	• AAPPO/HADS • ≥ 50% hair loss vignette • Literature review/study sponsor materials • Patient interview data
0–10% scalp hair loss (SALT 0–10)	• AAPPO/HADS • ≥ 50% hair loss vignette • Literature review/study sponsor materials • Patient interview data
Caregiver of adolescent with ≥ 50% scalp hair loss	• Literature review/study sponsor materials • Caregiver interview data

*AAPPO* alopecia areata patient priority outcomes questionnaire, *HADS* Hospital anxiety and depression scale, *SALT* severity of alopecia tool

^a^Eyebrow and eyelash hair loss vignette included after interview phase based on feedback

### Health state vignette development

The vignette development combined quantitative data from validated PRO measures, findings from a literature review and qualitative data from interviews with people affected by AA. The literature review was performed in November 2021, followed by the clinical trial analysis in May 2022. Qualitative interviews with patients with AA, caregivers and health care professionals (HCPs) to develop and refine the vignettes were conducted between June and July 2022. TTO interviews with the UK public were conducted between October and November 2022.

#### Targeted literature review

Targeted literature searches were conducted via OVID, a digital search engine, using EMBASE and Medline databases with title and abstract searches (conducted 16th November 2021). No date limits were applied. Studies were included if they reported qualitative or quantitative data on patient or carer symptoms or HRQoL impacts of AA (other forms of hair loss were excluded). Study populations included patients aged ≥ 12 and caregivers aged ≥ 18. Searches explored the HRQoL burden of AA in adult and adolescent patients and caregivers (Online Resource 1). Findings for both searches were summarised based on key themes to support the vignette descriptions. Differences by extensiveness of hair loss were highlighted.

#### Clinical trial data analysis

AA treatment clinical trial ALLEGRO-2b/3 [29] and real-world [30] PRO data were analysed to inform the descriptions of patient health states. Both studies recruited patients with AA with at least 50% hair loss (i.e. SALT ≥ 50). Participants in both studies completed the hospital anxiety and depression scale (HADS) [31] and alopecia areata patient priority outcomes (AAPPO) questionnaire [32]. Participants were grouped by SALT categories (e.g. SALT 0–10, SALT 11–20, SALT 21–49, SALT 50–100). Item level responses for HADS and AAPPO items were summarised using frequencies and proportions for each SALT sub-group.

The most frequently reported response options for each item of the AAPPO and HADS from the trial data were used as the basis for drafting the patient health state vignettes. When there was no clear modal value, the distribution of responses was considered. The selection of items for the vignettes also considered the degree of conceptual overlap between items, patient relevance and complexity. Language from the AAPPO and HADS items was used in the draft vignettes with some minor amendments. Additional summary analyses were also undertaken to explore differences by (1) age group (adolescents and adults), (2) levels of eyebrow loss (AAPPO item 2) and (3) levels of eyelash loss (AAPPO item 3).

#### Qualitative interviews

Semi-structured interviews were conducted with adult (aged ≥ 18 years) and adolescent (aged 12–17 years) patients with AA and caregivers of adolescents (aged 12–17 years) with AA from the UK. Participants were recruited via a specialist recruitment agency using various methods (e.g. online panel and social media). The purpose of the caregiver interviews was to provide evidence to develop caregiver vignette content. Participants with AA were eligible if they could provide confirmation of AA diagnosis, had experienced SALT ≥ 50 hair loss and were willing to consent to take part in a recorded semi-structured interview. Patients with current hair loss SALT ≥ 50 were required to have recent experience with systemic treatment for AA or an interest in receiving systemic treatment. Caregivers were eligible if they were the parent or main caregiver of a child with AA aged 12–17 years. Adolescents provided informed assent combined with parental consent prior to participation. Adolescents and caregivers were interviewed in the same interview. Caregivers had the option to chaperone the adolescent when interviewed. Interviews followed a standardised semi-structured interview guide, allowing interviewers to probe for further detail on topics of interest and explore spontaneous topics when mentioned.

The first phase of interviews explored the impact of AA on the HRQoL of adult and adolescent patients and their caregivers. Topics covered included the extent of hair loss experienced, other physical symptoms and impacts on daily activities, relationships and emotional wellbeing. Interviews were transcribed, anonymised and analysed using a content analysis approach. Specific quotes were examined to identify key terms used to describe the severity of the symptomatic and HRQoL burden. Data were coded by experienced researchers, supported by MAXQDA2020 software. A post-coding comparison and reconciliation was conducted, with all codes compared, discussed and reconciled wherever differences occurred. Once agreement between coders was reached, one coder analysed the remaining transcripts. These data were used to support vignette content.

In the second phase, cognitive debrief interviews were conducted with new participants to assess the accuracy of the draft vignettes and whether they represented the typical patient/caregiver experience. No adolescent patients were included in this phase as no adolescent-specific vignette was developed (further detail provided in results section). HCPs currently treating patients with AA were also invited to review the accuracy of the draft vignettes. HCPs were identified from recently published peer-reviewed literature. Where there was disagreement between HCPs and patient/caregivers, the view of the patient/caregiver was given greater weight. Vignettes were then revised and finalised.

### Health state valuation

A representative sample of the UK population in terms of age, gender and ethnicity, based on census data (2011) available at the time of recruitment [33], was recruited to complete the TTO interviews via a specialist recruitment agency. Participants were eligible if they were aged ≥ 18, resident in the UK, fluent in English and willing to provide consent. Participants also completed background questionnaires. Interviews were completed online using the Zoom platform with experienced moderators (*N* = 4). Participants rated their own current HRQoL using the EQ-5D-5L.

During interviews, participants read through each vignette and rated them using the Visual analogue scale (VAS). The VAS rating scale ranges from 0 (worst health possible) to 100 (full health). Participants also rated a ‘dead’ state on the VAS. The responses were rescaled where the dead state was scored as a value other than zero using the formula below, where V′ is the rescaled VAS value, *V* is the original VAS value and VDead is the value given to the Dead state.

$${V}{\prime}=\left(\frac{V-{V}_{Dead}}{100-{V}_{Dead}}\right)*100$$

In the TTO valuation task, participants read the vignettes again, and asked to imagine they were in each health state. They were then asked to choose between remaining in the health state without improvement for ten years (followed by death), or to live a shorter number of years in “full health” followed by death. The process incorporates a ‘ping-pong’ approach where high and low values are alternately presented [34] with participants trading increments of 6 months in full health to avoid living in the health state until they reach the point of indifference, where the participant believes the two prospects are the same. If participants reported a preference for zero years of full health over ten years in a health state, this indicates that they believe the state to be worse than being dead. At this point, the lead-time TTO method was used, in which each health state was preceded by ten years of full health. This allows participants to quantify how much worse than death they believe a health state to be [35]. The order the vignettes were presented was pseudo-randomised. All valuation data were summarised using descriptive statistics. Utilities were calculated using the below formula, where the utility of a health state (Ui) is the point of indifference (*X*) divided by the maximum time horizon (*T*), i.e. 10 years.

$${\text{U}}_{{\text{i}}} = \frac{X}{T}$$

A pilot study (*N* = 20) was undertaken to assess the face validity of valuations and vignette comprehension. Following this review, the full sample was recruited and analysed. A sensitivity analysis was conducted to explore how the results were affected by removing participants who may not have understood the task. Three exclusion criteria were used:

1. Participant who values the mildest patient state as worse than the most severe state
2. Participant who trades life and subsequently values all health states the same (e.g. all health states valued at 0.975)
3. Participant who TTO interviewers noted as experiencing comprehension difficulties or being disengaged with the TTO task

Analyses were performed with R 4.1.2 [36] and the R package ‘eq5d’ [37].

## Results

### Development of health states

#### Literature review

The review of patient burden identified 765 hits, with 35 considered eligible for review. The review highlighted the burden and impact of scalp hair loss, as well as eyebrow and eyelash hair loss [10, 38]. Other symptoms include patchy regrowth, headaches, itchiness, eye irritation, running nose, burning pain and bleeding [9]. AA is associated with higher rates of anxiety and depression [6, 13, 39], and feelings of fear, embarrassment, worry, frustration and self-consciousness [10, 11, 38, 40]. The psychological impact can also lead to a withdrawal from daily and social activities [10, 15, 41]. There is evidence that the symptomatic and psychosocial burden is greater with more extensive hair loss [6, 9, 12, 42]. No clear differences in the experience of adults and adolescents were reported.

The review of caregiver burden in AA identified 70 hits, with 6 manuscripts reviewed in full. The caregivers/parents of patients with AA can experience a significant HRQoL impact [13, 43–45]. Caregivers of patients with AA had worse HRQoL than caregivers of people with other skin conditions [45]. One study reported that a greater degree of patient hair loss was associated with a more significant HRQoL impact on the caregiver [46]. Emotional and psychosocial domains of HRQoL were the most frequently and severely impacted [43, 46].

#### Trial analysis

The results from the trial analyses are reported in Online Resource 2 and Online Resource 3. The AAPPO data highlighted the emotional burden of AA, especially in patients with more extensive hair loss. The frequency of responses for the HADS depression items suggested that very few patients experience depressive symptoms. However, most patients experience some symptoms of anxiety, more frequently so in those with more extensive hair loss. Stratified analyses comparing adults vs. adolescents and those with vs. without eyebrow and eyelash hair loss showed no clear differences in responses to the AAPPO and HADS (data not shown).

#### Concept elicitation interviews with patients and caregivers

Exploratory qualitative interviews were conducted with three adult patients, three adolescent patients and five caregivers (Online Resource 4). Additional detail is provided on the caregiver data in this section as these were the primary source for informing caregiver vignette development. Patients had experienced scalp hair loss ranging from 0 to 95%. Patients described self-consciousness, anxiety/worry and sadness/depression. Other less frequently reported emotional impacts included stress, loss of confidence, frustration/anger, embarrassment, loss of identity and loneliness. There was some evidence that feelings of anxiety and depression are more severe with more extensive hair loss.

“I can get…quite sad about it or I can get like quite irritated or in a bad mood…with people that are closest to me.” (Adult patient, hair loss 95–100%)

The most frequently reported emotional impacts experienced by caregivers included stress, anxiety/worry, frustration/anger and sadness/depression. The emotional impact on caregivers was ongoing because of the unpredictable nature of the condition.

“It was very stressful for me so at that time I …. had low appetite at that time. It was a year back, and I was sleepless.” (Caregiver, child hair loss SALT ≥ 50)”

Some adult patients reported sleep disturbances caused by anxiety/worry associated with AA. Patients reported an impact on their ability to meet new people and engage in romantic relationships. Additional time spent on concealments/routines was reported, which impacted usual activities. Patients reported withdrawal from work/school and social activities and avoidance of physical activities or hobbies where concealments could not be used.

“I’ve basically not met anyone new for the past year—I don’t want to take pictures of me like this—at my worst” (Adult patient, hair loss 50–94%)

The most frequent impacts on usual activities reported by caregivers included time spent assisting with concealments, work impacts, sleep disturbance, and relationships.

“I had to be signed off work because I was so upset.” (Caregiver, child hair loss SALT ≥ 50)

Caregivers reported impacts on relationships with partners and other children because of the additional time spent caring for their child with AA. Caregivers also reported emotional impacts on family members.

“It probably was causing a bit of stress on the family” (Caregiver, child hair loss SALT ≥ 50)

The findings from the exploratory interviews were incorporated into the vignettes. The qualitative and quantitative data that were used to develop the vignettes suggested very few reliable differences between the experience of adults and adolescents with AA. Therefore, it was concluded that it would not be necessary to recruit additional adolescents to participate in cognitive debrief interviews as the vignettes would be valid representations of both adult and adolescent experiences.

#### Cognitive debrief interviews

Five different adult patients and four HCPs reviewed the draft patient vignettes. Patients had experienced varying levels of hair loss (Online Resource 5). The HCPs were all consultant dermatologists, treating between 15 and 350 patients with AA per year. Overall, patients and HCPs agreed that the vignettes captured most HRQoL impacts associated with AA. There was consensus that the severity of the impact on social and physical activities, self-consciousness/embarrassment, frustration, confidence, sadness, enjoyment, tension and worry was understated. Patients also identified impacts not described in the vignettes, including the effects on sleep, loss of identity and discomfort.

Other minor suggested changes included adding more contextual information (e.g. social activities impacted), simplifying content and improving readability (Table 2). Patients and HCPs also highlighted a lack of detail regarding eyebrow and eyelash hair loss and how this impacts a range of HRQoL domains, including confidence, social activities, frustration, worry and discomfort. An additional health state describing SALT ≥ 50 scalp hair loss with eyebrow & eyelash loss was therefore developed, and statements were added to other health states to confirm eyelash/eyebrow hair loss was unimpacted.

**Table 2:** Summary of revisions to patient and caregiver vignettes

Content change	Source	Original text	Final text
Patient vignettes			
Impact on eyebrow and eyelash hair loss and additional health state describing SALT ≥ 50 + eyebrow and eyelash hair loss	Patient and HCP	None	*SALT 0–10, SALT 11–20, SALT 21–49, SALT* ≥ *50*: ‘You have normal eyebrows and eyelashes.’ *SALT* ≥ *50* + *eyebrow & eyelash hair loss:* ‘Most or all of your eyebrow and eyelash hair is also missing’
Increased frequency of impact on physical activity	Patient and HCP	*SALT 0–10: ‘*You do not limit your participation in physical activities or exercise because of your hair loss at all.’ *SALT 11–20, SALT 21–49, SALT* ≥ *50*: ‘You limit your participation in physical activities or exercise because of your hair loss a little/quite a bit.’	*SALT 0–10:* ‘You rarely don’t take part in physical activities or exercise because of your hair loss.’ *SALT 11–20, SALT 21–49, SALT* ≥ *50, SALT* ≥ *50* + *eyebrow & eyelash hair loss*: ‘Occasionally/ sometimes/most of the time you do not take part in physical activities or exercise because of your hair loss.’
Increased frequency of impact on social activities. Contextualised impact on social activities	Patient and HCP	*SALT 0–10: ‘*You do not limit your social interactions because of your hair loss at all.’ *SALT 11–20, SALT 21–49, SALT* ≥ *50*: ‘You limit your social interactions because of your hair loss a little/quite a bit.’	*SALT 0–10:* ‘You do not limit your social interactions with friends and family because of your hair loss and you do not find it difficult to meet new people.’ *SALT 11–20, SALT 21–49, SALT* ≥ *50, SALT* ≥ *50* + *eyebrow & eyelash hair loss*: ‘You limit your social interactions with friends and family because of your hair loss some of the time/quite a lot/a lot and you occasionally/often/frequently find it difficult to meet new people.’
Increased frequency of impact on confidence	Patient and HCP	*All health states: ‘*You very rarely/rarely/sometimes lack confidence in your appearance because of your hair loss.’	*All health states: ‘*You rarely/sometimes/often/frequently/very frequently lack confidence in your appearance because of your hair loss.’
Increased frequency of frustration and added feelings of anger	Patient and HCP	*All health states: ‘*You never/very rarely/rarely/sometimes feel frustrated about your hair loss.’	*All health states: ‘*You rarely/often/frequently/very frequently feel frustrated and angry about your hair loss.’
Increased frequency of impact on self-consciousness/embarrassment	Patient and HCP	*All health states: ‘*You very rarely/rarely/sometimes feel self-conscious or embarrassed about your hair loss.’	*All health states: ‘*You rarely/occasionally/often/frequently/very frequently feel self-conscious or embarrassed about your hair loss.’
Increased frequency and severity of sadness/depression	Patient and HCP	*All health states:* ‘You very rarely/rarely/sometimes feel sad about your hair loss.’	*SALT 0–10, SALT 11–20:* ‘You rarely/occasionally feel sad and depressed about your hair loss’ *SALT 21–49, SALT* ≥ *50, SALT* ≥ *50* + *eyebrow & eyelash hair loss*: ‘You often/frequently feel sad and depressed about your hair loss. Sometimes the sadness and depression are severe.’
Increased frequency of impact on feeling tense/wound up and reworded to anxiety and stress	Patient and HCP	*All health states:* ‘You occasionally feel tense or wound up’	*All health states:* ‘You occasionally/sometimes/often/frequently/very frequently feel anxious and stressed’
Increased frequency of worrying	Patient and HCP	*All health states:* ‘Worrying thoughts very rarely/occasionally go through your mind.’	*All health states:* ‘Worrying thoughts occasionally/sometimes/often/frequently/very frequently go through your mind.’
Added impact on sleep/tiredness	Patient	None	*All health states:* ‘Your sleep is very rarely/rarely/occasionally/sometimes disturbed…and you very rarely/rarely/occasionally/sometimes feel tired’
Added impact on mild discomfort	Patient	None	*SALT 0–10, SALT 11–20, SALT 21–49, SALT* ≥ *50:* ‘You rarely/occasionally/sometimes experience mild discomfort on your skin or nails.’ *SALT* ≥ *50* + *eyebrow & eyelash hair loss: ‘*You sometimes experience mild discomfort on your skin, nails or around your eyes.’
Added impact on loss of identity	Patient	None	*SALT 0–10, SALT 11–20: ‘*You rarely/occasionally do not feel like yourself.’ *SALT 21–49, SALT* ≥ *50, SALT* ≥ *50* + *eyebrow & eyelash hair loss:* ‘You go through periods of not feeling like yourself.’
Caregiver vignette			
Contextualised support provided	Caregiver	‘You need to spend some time on most days supporting your family member (e.g., helping to conceal hair loss)’	‘You need to spend some time on most days supporting your family member (e.g., helping to conceal hair loss, providing emotional or social support)’
Increased impact on usual activities	Caregiver	‘You are able to do your usual activities, such as going to work, socialising and leisure activities’	‘Most of the time you are able to do your usual activities, such as going to work, socialising and leisure activities’
Decreased frequency of frustration	Caregiver	‘You frequently feel frustrated about your family member’s condition’	‘You occasionally feel frustrated about your family member’s condition’
Decreased severity of sadness/depression	Caregiver	‘You sometimes feel sad or depressed’	‘You sometimes feel sad’

*AAPPO* alopecia areata patient priority outcomes questionnaire, *SALT* severity of alopecia tool, *HCP* healthcare professional, *SALT ≥ 50* 50–100% scalp hair loss, *SALT 21–49* 21–49% scalp hair loss, *SALT 11–20* 11–20% scalp hair loss, *SALT 0–10* 0–10% scalp hair loss

Interviews were conducted with five caregivers of adolescents with AA (Online Resource 5). Caregivers largely endorsed the vignette content as accurate. However, the severity of frustration and sadness/depression was overstated and the impact on usual activities understated.

The final vignettes were refined based on the interview feedback (Online Resource 6). Feedback from the pilot TTO interviews (*N* = 20) revealed no concerns over face validity or issues with vignette comprehension. Subsequently, no changes were made to the vignettes and the pilot data were included in the final sample.

### Health state valuations

#### Sample characteristics

A total of *N* = 120 members of the UK public participated; demographic characteristics are listed in Table 3***.*** The sample was broadly consistent with the most recent UK census data (2021) available at the time of reporting [47].

**Table 3:** Sample demographic characteristics of UK general public completing time trade off valuation task

Characteristic	UK sample (*N* = 120)	UK 2021 census data^a^
Age,		
Mean (SD)	41.1 (15.2)	40
Range	18.0–77.0	
Gender		
Male	60 (50%)	49%
Female	60 (50%)	51%
Ethnicity		
White	102 (85%)	82%
Mixed or multiple ethnicity	5 (4.2%)	
Asian or Asian British	4 (3.3%)	
Black, African, Caribbean or Black British	9 (7.5%)	
Other ethnic group	0 (0%)	
Prefer not to answer	0 (0%)	
Country		
England	109 (91%)	
Wales	5 (4%)	
Scotland	6 (5%)	
Northern Ireland	0 (0%)	
Employment		
Employed full-time	58 (48%)	
Employed part-time	29 (24%)	
Self employed	7 (5.8%)	
Stay at home or full-time carer	3 (2.5%)	
Retired	8 (6.7%)	
Seeking work/unemployed	2 (1.7%)	
Long-term sick leave	1 (0.8%)	
Student	12 (10%)	
Other	0 (0%)	
Lives with self-reported long-term condition		
Yes	21 (18%)	
No	99 (82%)	
Prefer not to answer	0 (0%)	

SD standard deviation, UK United Kingdom

^a^Figures based on data from the 2021 United Kingdom national census (Office for National Statistics, https://www.ons.gov.uk/census)

#### Health state valuations

VAS ratings (Table 4) ranged from 77.6 (SALT 0–10) to 39.1 (SALT 50–100 + eyebrow and eyelash loss). The perceived HRQoL burden was higher for states with greater scalp hair loss. UK public TTO utility weights for the patient health states range from 0.502 (SALT 50–100 + eyebrow and eyelash loss) to 0.919 (SALT 0–10). The mean caregiver health state UK public TTO utility weight is 0.882. TTO utility weights mirror the pattern observed for the VAS valuations, with a higher perceived HRQoL impact observed where the level of hair loss was higher. The addition of eyebrow and eyelash loss also increased the perceived HRQoL burden.

**Table 4:** Rescaled visual analogue scale and time trade off health state valuations

Health states	Visual analogue scale ratings			TTO utility weights		
*N* = 120	Mean (SD)	Range	95% CI	Mean (SD)	Range	95% CI
SALT 0–10	77.6 (13.6)	20.0–100.0	75.1–80.0	0.919 (0.119)	0.175–1.000	0.898–0.941
SALT 11–20	65.8 (16.0)	20.0–95.0	62.9–68.7	0.853 (0.227)	− 1.000–0.975	0.812–0.894
SALT 21–49	53.4 (17.4)	1.2–95.0	50.3–56.6	0.703 (0.312)	− 1.000–0.975	0.647–0.759
SALT 50–100	44.8 (19.7)	− 14.3–95.0	41.2–48.4	0.554 (0.468)	− 1.000–0.975	0.471–0.638
SALT 50–100 + eyebrow and eyelash loss	39.1 (20.0)	− 22.0–90.0	35.5–42.8	0.502 (0.469)	− 1.000–0.975	0.418–0.586
*N* = 57^a^						
Caregiver	70.7 (13.2)	33.3–95.0	67.2–74.2	0.882 (0.128)	0.375–1.000	0.849–0.915

*SD* standard deviation, *SALT* severity of alopecia tool, *TTO* time trade off

^a^The caregiver vignette was finalised and introduced into valuation interviews after fieldwork with patient health states was initiated resulting in a reduced sample size

#### Sensitivity analysis

Six participants were excluded based on the exclusion criteria. No significant changes to the individual health state utilities were observed after exclusion of these data points (Online Resource 7). However, the range between the mildest and most severe health states was slightly larger compared to the full sample.

## Discussion

This study aimed to elicit UK public utilities describing AA states defined by SALT scores. UK public utilities were elicited for five patient health states and one caregiver health state. Vignettes were developed using multiple data sources, including a literature review and PRO data from a large clinical trial. Detailed qualitative data were also collected and rounds of review were undertaken to adjust the vignettes. This approach is consistent with guidance from the NICE on best practice for developing vignette studies [26, 28].

Utilities elicited in this study describe the HRQoL impact caused by AA for patients and caregivers of adolescents with AA. VAS ratings and TTO weights were lower for states with greater hair loss [49]. The range in utility values (0.502–0.919) was similar to those observed in other dermatological conditions, such as atopic dermatitis (0.42–0.91) [49, 50], acne vulgaris (0.72–0.94) [51] and chronic spontaneous urticaria (0.601–0.859) [52–54]. The utility value for the least extensive hair loss state (SALT 0–10 = 0.919), was comparable to UK EQ-5D population norms [48].

Utilities elicited using the EQ-5D are preferred by many decision-makers because it provides standardisation of methods, allowing for a ‘common currency’ of expected benefits across diseases/indications and the efficient allocation of resources. However, standardisation is only effective and equitable if the measure (i.e. the EQ-5D) is appropriate across all its applications. It may, therefore, be flawed if it is unable to measure the burden of a disease, such as AA, accurately.

As noted above, NICE recognise the limitations of the EQ-5D in skin conditions. EQ-5D utility values for AA published in the literature show a narrow range of values between mild and severe health states [24, 25]. One study, among European patients, reports EQ-5D scores ranging from mild (0.89) to severe AA (0.77) [55]. Less differentiation was reported between mild (0.95) and severe (0.87) AA in a similar study of US patients [25]. The present study found that the differences between AA states are much larger.

It is possible that the vignette approach is more sensitive than the EQ-5D for capturing the full HRQoL impact of hair loss in AA. The difference in utility weights between the most extensive hair loss state and the least in this study is large (approximately 0.40). This is likely related to the substantial psychosocial effect that people with AA experience. Indeed, hair loss due to AA can lead to a change in self-identity and loss of self-esteem, with some patients likening the experience to devastation or grief [15, 56, 57]. Patients also report withdrawal from daily activities, leading to a reduction in social interactions, participation in physical activities and engagement in romantic and intimate relationships [1, 13, 56, 58, 59]. The wide range in utility values observed is consistent with the notion that hair plays an important role in various aspects of HRQoL. Future work should explore to what extent the EQ-5D is insensitive to the impact of AA.

Vignette studies rest on the content validity of the vignettes used to elicit utilities [28]. A key strength of this study was the use of qualitative and quantitative data sources to develop and validate the vignettes. The use of data from two validated PRO measures generated in a clinical trial, one AA-specific (AAPPO) and one generic anxiety and depression measure (HADS), combined with a targeted literature review, provided a strong basis to describe the burden of AA. Vignettes were refined based on the qualitative data obtained from patients with varying levels of hair loss, reflecting the full range of hair loss described in the vignettes.

There are some limitations to be considered in the data sources used to develop the vignettes. The protocol of the clinical trial which informed the draft vignettes excluded individuals with psychiatric conditions (i.e. suicidal ideation, clinically significant depression). This may have led to an underestimation of the mental health impacts described in the draft vignettes. Limitations to the patient sample recruited to participate in qualitative interviews should also be considered. All participants in the debrief interviews were adults and female, which could have biased the review of the vignettes. It is also possible that the views of the patient sample may not be representative of the wider population of patients with AA and that new concepts may have emerged if additional interviews were conducted. Finally, the vignettes do not account for the heterogeneity in patient experience with the same extent of hair loss.

## Conclusion

This study elicited societal utility values for health states describing the HRQoL burden of AA for patients and caregivers. The perceived HRQoL impact was greater for health states describing more extensive hair loss. Health state vignettes were developed using multiple sources of qualitative and quantitative evidence to ensure a robust evidence base. The utilities elicited enable economic evaluations of current and future treatments that could successfully treat AA.

### Supplementary Information

Below is the link to the electronic supplementary material.

Supplementary file1 (PDF 112 kb)

Supplementary file2 (PDF 202 kb)

Supplementary file3 (PDF 210 kb)

Supplementary file4 (PDF 139 kb)

Supplementary file5 (PDF 140 kb)

Supplementary file6 (PDF 185 kb)

Supplementary file7 (PDF 145 kb)

## Data Availability

The data that support the findings of this study are available from the corresponding author upon reasonable request.
