# Supplementary material for: Estimation of health utility values for alopecia areata
Source: Qual Life Res. 2024 Mar 29;33(6):1581–92. doi: 10.1007/s11136-024-03645-9 (PMC11116246; doi:10.1007/s11136-024-03645-9)
Supplement: Supplementary file 1 — Supplementary file1 (PDF 112 kb) [file 11136_2024_3645_MOESM1_ESM.pdf]

Article title: Estimation of health utility values for alopecia areata

Journal name: Quality of Life Research

Author names: Daniel Aggio, Caleb Dixon, Ernest H. Law, Rowena Randall, Thomas Price, Andrew Lloyd

Corresponding Author: Daniel Aggio (Daniel.Aggio@acasterlloyd.com); Acaster Lloyd Consulting Ltd. 8th Floor, Lacon House, 84 Theobalds Road, London WC1X 8NL

Online Resource 1. Targeted literature review search strings

Search 1 (patient burden) search terms
Alopecia Areata [title/abstract] OR Total alopecia areata [title/abstract] OR Alopecia totalis [title/abstract] OR Alopecia universalis [title/abstract] OR Patchy Alopecia Areata [title/abstract] OR Ophiasic Alopecia Areata OR Ophiasis [title/abstract] OR Universal Alopecia Areata [title/abstract]<br>AND<br>Quality of life [title/abstract] OR QOL [title/abstract] OR HRQoL [title/abstract] OR Burden [title/abstract] OR wellbeing [title/abstract] OR PRO [title/abstract] OR patient reported [title/abstract] OR clinician reported [title/abstract] OR ClinRO [title/abstract] OR Severity of alopecia tool [title/abstract] OR severity of alopecia areata tool [title/abstract] OR AAPPO OR alopecia areata patient priority outcomes instrument [title/abstract] OR AASIS [title/abstract] OR alopecia areata symptom impact scale [title/abstract] OR AAQ [title/abstract] OR alopecia areata quality of life [title/abstract] OR AA-QLI [title/abstract] OR SF-12 [title/abstract] OR short-form 12 [title/abstract] OR SF-36 [title/abstract] OR short-form 36 [title/abstract] OR GAD* [title/abstract] OR generalized anxiety disorder assessment [title/abstract] OR PHQ* [title/abstract] OR patient health questionnaire [title/abstract] OR activities of daily living [title/abstract] OR ADL* [title/abstract]

Search 2 (caregiver burden) search terms
Alopecia Areata [title/abstract] OR Total alopecia areata [title/abstract] OR Alopecia totalis [title/abstract] OR Alopecia universalis [title/abstract] OR Patchy Alopecia Areata [title/abstract] OR Ophiasic Alopecia Areata OR Ophiasis [title/abstract] OR Universal Alopecia Areata [title/abstract]<br>AND<br>Carer [title/abstract] or caregiver [title/abstract] or parent* [title/abstract] or father [title/abstract] or mother [title/abstract] or family [title/abstract] or caretaker [title/abstract] or sibling* [title/abstract] or brother* [title/abstract] or sister* [title/abstract]<br>AND<br>Quality of life [title/abstract] OR QOL [title/abstract] OR HRQoL [title/abstract] OR Burden [title/abstract] OR wellbeing [title/abstract] title/abstract] OR Severity of alopecia tool [title/abstract] OR severity of alopecia areata tool [title/abstract] OR AAPPO OR alopecia areata patient priority outcomes instrument [title/abstract] OR AASIS [title/abstract] OR alopecia areata symptom impact scale [title/abstract] OR AAQ [title/abstract] OR alopecia areata quality of life [title/abstract] OR AA-QLI [title/abstract] OR SF-12 [title/abstract] OR short-form 12 [title/abstract] OR SF-36 [title/abstract] OR short-form 36 [title/abstract] OR GAD* [title/abstract] OR generalized anxiety disorder assessment [title/abstract] OR PHQ* [title/abstract] OR patient health questionnaire [title/abstract] OR activities of daily living [title/abstract] OR ADL* [title/abstract] OR family dermatology quality of life index [title/abstract] OR FDLQI [title/abstract] OR caregiver reported [title/abstract] OR parent reported [title/abstract]
