# Supplementary material for: Estimation of health utility values for alopecia areata
Source: Qual Life Res. 2024 Mar 29;33(6):1581–92. doi: 10.1007/s11136-024-03645-9 (PMC11116246; doi:10.1007/s11136-024-03645-9)
Supplement: Supplementary file 2 — Supplementary file2 (PDF 202 kb) [file 11136_2024_3645_MOESM2_ESM.pdf]

Article title: Estimation of health utility values for alopecia areata

Journal name: Quality of Life Research

Author names: Daniel Aggio, Caleb Dixon, Ernest H. Law, Rowena Randall, Thomas Price, Andrew Lloyd

Corresponding Author: Daniel Aggio (Daniel.Aggio@acasterlloyd.com); Acaster Lloyd Consulting Ltd. 8th Floor, Lacon House, 84 Theobalds Road, London WC1X 8NL

Online Resource 2. AAPPO item responses among all patients (n=636) from AA treatment clinical trial ALLEGRO-2b/3 by SALT scores

Characteristic	*SALT <= 10, N = 530*[1]	*SALT > 10 & <=20, N = 213*[1]	*SALT > 20 & <50, N = 542*[1]	*SALT >= 50 to 100, N = 3,919*[1]	*SALT >= 50 to 99, N = 2,404*[1]	*SALT 100, N = 1,515*[1]
**How would you describe the current amount of hair loss you have on your eyebrows? (AAPPO item 2)**						
No eyebrow hair loss	331 (62%)	98 (46%)	225 (42%)	617 (16%)	569 (24%)	48 (3.2%)
A little hair loss	121 (23%)	48 (23%)	121 (22%)	410 (10%)	367 (15%)	43 (2.8%)
Moderate hair loss	42 (7.9%)	41 (19%)	88 (16%)	378 (9.6%)	303 (13%)	75 (5.0%)
A great deal of hair loss	35 (6.6%)	24 (11%)	93 (17%)	928 (24%)	660 (27%)	268 (18%)
Complete (no eyebrow hair)	1 (0.2%)	2 (0.9%)	15 (2.8%)	1,585 (40%)	504 (21%)	1,081 (71%)
Missing	0	0	0	1	1	0
**How would you describe the current amount of hair loss you have of your eyelashes? (AAPPO item 3)**						
No eyelash hair loss	339 (64%)	101 (47%)	254 (47%)	873 (22%)	778 (32%)	95 (6.3%)
A little hair loss	110 (21%)	47 (22%)	109 (20%)	456 (12%)	358 (15%)	98 (6.5%)
Moderate hair loss	47 (8.9%)	31 (15%)	64 (12%)	409 (10%)	276 (11%)	133 (8.8%)
A great deal of hair loss	30 (5.7%)	28 (13%)	87 (16%)	694 (18%)	473 (20%)	221 (15%)

Characteristic	*SALT <= 10, N = 530*[1]	*SALT > 10 & <=20, N = 213*[1]	*SALT > 20 & <50, N = 542*[1]	*SALT >= 50 to 100, N = 3,919*[1]	*SALT >= 50 to 99, N = 2,404*[1]	*SALT 100, N = 1,515*[1]
Complete (no eyelash hair)	4 (0.8%)	6 (2.8%)	28 (5.2%)	1,486 (38%)	518 (22%)	968 (64%)
Unknown	0	0	0	1	1	0
**Over the past week, how often did you feel self-conscious about your hair loss? (AAPPO item 5)**						
Never	281 (53%)	80 (38%)	166 (31%)	899 (23%)	563 (23%)	336 (22%)
Rarely	128 (24%)	60 (28%)	157 (29%)	955 (24%)	570 (24%)	385 (25%)
Sometimes	84 (16%)	53 (25%)	132 (24%)	1,059 (27%)	630 (26%)	429 (28%)
Often	24 (4.5%)	14 (6.6%)	61 (11%)	599 (15%)	357 (15%)	242 (16%)
Always	12 (2.3%)	6 (2.8%)	25 (4.6%)	406 (10%)	284 (12%)	122 (8.1%)
Missing	1	0	1	1	0	1
**Over the past week, how often did you feel embarrassed about your hair loss? (AAPPO item 6)**						
Never	333 (63%)	95 (45%)	219 (40%)	1,329 (34%)	812 (34%)	517 (34%)
Rarely	113 (21%)	68 (32%)	150 (28%)	1,006 (26%)	600 (25%)	406 (27%)
Sometimes	62 (12%)	36 (17%)	106 (20%)	850 (22%)	498 (21%)	352 (23%)
Often	19 (3.6%)	12 (5.6%)	52 (9.6%)	447 (11%)	282 (12%)	165 (11%)
Always	2 (0.4%)	2 (0.9%)	14 (2.6%)	285 (7.3%)	212 (8.8%)	73 (4.8%)
Missing	1	0	1	2	0	2
**Over the past week, how often did you feel sad about your hair loss? (AAPPO item 7)**						
Never	319 (60%)	93 (44%)	211 (39%)	1,100 (28%)	676 (28%)	424 (28%)

Characteristic	*SALT <= 10, N = 530*[1]	*SALT > 10 & <=20, N = 213*[1]	*SALT > 20 & <50, N = 542*[1]	*SALT >= 50 to 100, N = 3,919*[1]	*SALT >= 50 to 99, N = 2,404*[1]	*SALT 100, N = 1,515*[1]
Rarely	129 (24%)	63 (30%)	162 (30%)	1,047 (27%)	646 (27%)	401 (26%)
Sometimes	64 (12%)	42 (20%)	106 (20%)	993 (25%)	565 (24%)	428 (28%)
Often	16 (3.0%)	14 (6.6%)	50 (9.2%)	504 (13%)	324 (13%)	180 (12%)
Always	1 (0.2%)	1 (0.5%)	12 (2.2%)	274 (7.0%)	193 (8.0%)	81 (5.4%)
Missing	1	0	1	1	0	1
**Over the past week, how often did you feel frustrated about your hair loss? (AAPPO item 8)**						
Never	343 (65%)	102 (48%)	211 (39%)	1,189 (30%)	731 (30%)	458 (30%)
Rarely	96 (18%)	56 (26%)	154 (28%)	988 (25%)	597 (25%)	391 (26%)
Sometimes	75 (14%)	41 (19%)	122 (23%)	954 (24%)	573 (24%)	381 (25%)
Often	13 (2.5%)	12 (5.6%)	43 (7.9%)	531 (14%)	323 (13%)	208 (14%)
Always	3 (0.6%)	2 (0.9%)	12 (2.2%)	256 (6.5%)	179 (7.4%)	77 (5.1%)
Missing	0	0	0	1	1	0
**Over the past week, how much did you limit your participation in outdoor activities because of your hair loss? (AAPPO item 9)**						
Not at all	464 (88%)	169 (79%)	412 (76%)	2,686 (69%)	1,627 (68%)	1,059 (70%)
A little	54 (10%)	28 (13%)	84 (15%)	620 (16%)	371 (15%)	249 (16%)
Moderately	10 (1.9%)	16 (7.5%)	35 (6.5%)	363 (9.3%)	226 (9.4%)	137 (9.0%)
A great deal	2 (0.4%)	0 (0%)	10 (1.8%)	198 (5.1%)	142 (5.9%)	56 (3.7%)
Completely	0 (0%)	0 (0%)	1 (0.2%)	51 (1.3%)	37 (1.5%)	14 (0.9%)
Missing	0	0	0	1	1	0

Characteristic	*SALT <= 10, N = 530*[1]	*SALT > 10 & <=20, N = 213*[1]	*SALT > 20 & <50, N = 542*[1]	*SALT >= 50 to 100, N = 3,919*[1]	*SALT >= 50 to 99, N = 2,404*[1]	*SALT 100, N = 1,515*[1]
**Over the past week, how much did you limit your exercise or other physical activity because of your hair loss? (AAPPO item 10)**						
Not at all	486 (92%)	167 (78%)	439 (81%)	2,866 (73%)	1,746 (73%)	1,120 (74%)
A little	36 (6.8%)	29 (14%)	70 (13%)	539 (14%)	331 (14%)	208 (14%)
Moderately	6 (1.1%)	15 (7.0%)	22 (4.1%)	294 (7.5%)	174 (7.2%)	120 (7.9%)
A great deal	0 (0%)	2 (0.9%)	8 (1.5%)	170 (4.3%)	116 (4.8%)	54 (3.6%)
Completely	2 (0.4%)	0 (0%)	3 (0.6%)	49 (1.3%)	36 (1.5%)	13 (0.9%)
Missing	0	0	0	1	1	0
**Over the past week, how much did you limit your interactions with others because of your hair loss? (AAPPO item 11)**						
Not at all	479 (90%)	171 (80%)	422 (78%)	2,799 (71%)	1,695 (71%)	1,104 (73%)
A little	44 (8.3%)	28 (13%)	85 (16%)	647 (17%)	405 (17%)	242 (16%)
Moderately	3 (0.6%)	11 (5.2%)	27 (5.0%)	303 (7.7%)	181 (7.5%)	122 (8.1%)
A great deal	3 (0.6%)	3 (1.4%)	8 (1.5%)	132 (3.4%)	97 (4.0%)	35 (2.3%)
Completely	1 (0.2%)	0 (0%)	0 (0%)	37 (0.9%)	25 (1.0%)	12 (0.8%)
Missing	0	0	0	1	1	0

[1]n (%)

AA, Alopecia Areata; AAPPO, Alopecia Areata Patient Priority Outcomes; SALT, Severity of Alopecia Tool

Note: AAPPO Item 1 (current scalp hair loss) and 4 (current hair loss on body) not included in analysis
