# Supplementary material for: Estimation of health utility values for alopecia areata
Source: Qual Life Res. 2024 Mar 29;33(6):1581–92. doi: 10.1007/s11136-024-03645-9 (PMC11116246; doi:10.1007/s11136-024-03645-9)
Supplement: Supplementary file 3 — Supplementary file3 (PDF 210 kb) [file 11136_2024_3645_MOESM3_ESM.pdf]

Article title: Estimation of health utility values for alopecia areata

Journal name: Quality of Life Research

Author names: Daniel Aggio, Caleb Dixon, Ernest H. Law, Rowena Randall, Thomas Price, Andrew Lloyd

Corresponding Author: Daniel Aggio (Daniel.Aggio@acasterlloyd.com); Acaster Lloyd Consulting Ltd. 8th Floor, Lacon House, 84 Theobalds Road, London WC1X 8NL

Online Resource 3. HADS item responses among all patients (n=636) from AA treatment clinical trial ALLEGRO-2b/3 by SALT score

Characteristic	*SALT <= 10, N = 242*[1]	*SALT > 10 & <=20, N = 105*[1]	*SALT > 20 & <50, N = 300*[1]	*SALT >= 50 to 100, N = 2,889*[1]	*SALT >= 50 to 99, N = 1,720*[1]	*SALT 100, N = 1,169*[1]
**I get a sort of frightened feeling like 'butterflies' in the stomach**						
Never	162 (67%)	70 (67%)	209 (70%)	1,713 (59%)	1,023 (60%)	690 (59%)
Occasionally	72 (30%)	31 (30%)	77 (26%)	979 (34%)	574 (33%)	405 (35%)
Often	5 (2.1%)	3 (2.9%)	10 (3.3%)	134 (4.7%)	84 (4.9%)	50 (4.3%)
Very often	3 (1.2%)	1 (1.0%)	3 (1.0%)	54 (1.9%)	37 (2.2%)	17 (1.5%)
Missing	0	0	1	9	2	7
**I can enjoy a good book or radio or TV program**						
Often	211 (87%)	82 (78%)	252 (84%)	2,282 (79%)	1,359 (79%)	923 (79%)
Sometimes	24 (9.9%)	21 (20%)	39 (13%)	474 (16%)	281 (16%)	193 (17%)
Not often	4 (1.7%)	1 (1.0%)	5 (1.7%)	79 (2.7%)	46 (2.7%)	33 (2.8%)
Very seldom	3 (1.2%)	1 (1.0%)	3 (1.0%)	45 (1.6%)	32 (1.9%)	13 (1.1%)
Missing	0	0	1	9	2	7
**I still enjoy the things I used to enjoy**						
Definitely	202 (83%)	81 (77%)	239 (80%)	1,984 (69%)	1,190 (69%)	794 (68%)
Not quite so much	34 (14%)	19 (18%)	53 (18%)	709 (25%)	415 (24%)	294 (25%)
Only a little	2 (0.8%)	3 (2.9%)	7 (2.3%)	143 (4.9%)	86 (5.0%)	57 (4.9%)
Hardly at all	4 (1.7%)	2 (1.9%)	1 (0.3%)	53 (1.8%)	29 (1.7%)	24 (2.1%)
**I feel restless as I have to be on the move**						

Characteristic	*SALT <= 10, N = 242*[1]	*SALT > 10 & <=20, N = 105*[1]	*SALT > 20 & <50, N = 300*[1]	*SALT >= 50 to 100, N = 2,889*[1]	*SALT >= 50 to 99, N = 1,720*[1]	*SALT 100, N = 1,169*[1]
Never	141 (58%)	59 (56%)	168 (56%)	1,435 (50%)	848 (49%)	587 (51%)
Not very much	79 (33%)	34 (32%)	111 (37%)	1,047 (36%)	627 (37%)	420 (36%)
Quite a lot	18 (7.4%)	12 (11%)	15 (5.0%)	322 (11%)	196 (11%)	126 (11%)
Very much indeed/definitely	4 (1.7%)	0 (0%)	5 (1.7%)	75 (2.6%)	46 (2.7%)	29 (2.5%)
Missing	0	0	1	10	3	7
**I can laugh and see the funny side of things:**						
As much as I always could	223 (92%)	89 (85%)	267 (89%)	2,370 (82%)	1,420 (83%)	950 (81%)
Not quite so much	14 (5.8%)	15 (14%)	25 (8.3%)	379 (13%)	215 (12%)	164 (14%)
Definitely not so much	2 (0.8%)	0 (0%)	3 (1.0%)	103 (3.6%)	67 (3.9%)	36 (3.1%)
Never	3 (1.2%)	1 (1.0%)	5 (1.7%)	37 (1.3%)	18 (1.0%)	19 (1.6%)
**I feel cheerful**						
Most of the time	189 (78%)	83 (79%)	210 (70%)	1,967 (68%)	1,179 (69%)	788 (67%)
Sometimes	46 (19%)	21 (20%)	83 (28%)	786 (27%)	457 (27%)	329 (28%)
Not often	3 (1.2%)	1 (1.0%)	4 (1.3%)	107 (3.7%)	60 (3.5%)	47 (4.0%)
Never	4 (1.7%)	0 (0%)	3 (1.0%)	29 (1.0%)	24 (1.4%)	5 (0.4%)
**I feel tense or wound up**						

Characteristic	*SALT <= 10, N = 242*[1]	*SALT > 10 & <=20, N = 105*[1]	*SALT > 20 & <50, N = 300*[1]	*SALT >= 50 to 100, N = 2,889*[1]	*SALT >= 50 to 99, N = 1,720*[1]	*SALT 100, N = 1,169*[1]
Never	96 (40%)	39 (37%)	128 (43%)	976 (34%)	600 (35%)	376 (32%)
From time to time, occasionally	125 (52%)	52 (50%)	134 (45%)	1,537 (53%)	896 (52%)	641 (55%)
A lot of the time	15 (6.2%)	9 (8.6%)	32 (11%)	284 (9.8%)	166 (9.7%)	118 (10%)
Most of the time	6 (2.5%)	5 (4.8%)	6 (2.0%)	92 (3.2%)	58 (3.4%)	34 (2.9%)
**I feel as if I am slowed down**						
Never	157 (65%)	54 (51%)	183 (61%)	1,598 (55%)	956 (56%)	642 (55%)
Sometimes	69 (29%)	44 (42%)	92 (31%)	1,056 (37%)	606 (35%)	450 (38%)
Very often	7 (2.9%)	4 (3.8%)	17 (5.7%)	180 (6.2%)	119 (6.9%)	61 (5.2%)
Nearly all the time	9 (3.7%)	3 (2.9%)	8 (2.7%)	55 (1.9%)	39 (2.3%)	16 (1.4%)
**I get sudden feelings of panic**						
Never	170 (70%)	76 (72%)	212 (71%)	1,800 (62%)	1,062 (62%)	738 (64%)
Not very often	62 (26%)	26 (25%)	75 (25%)	875 (30%)	538 (31%)	337 (29%)
Often	5 (2.1%)	2 (1.9%)	10 (3.3%)	159 (5.5%)	84 (4.9%)	75 (6.5%)
Very often	5 (2.1%)	1 (1.0%)	2 (0.7%)	46 (1.6%)	34 (2.0%)	12 (1.0%)
Unknown	0	0	1	9	2	7
**I look forward with enjoyment to things**						
As much as ever	207 (86%)	83 (79%)	242 (81%)	2,086 (72%)	1,259 (73%)	827 (71%)

Characteristic	*SALT <= 10, N = 242*[1]	*SALT > 10 & <=20, N = 105*[1]	*SALT > 20 & <50, N = 300*[1]	*SALT >= 50 to 100, N = 2,889*[1]	*SALT >= 50 to 99, N = 1,720*[1]	*SALT 100, N = 1,169*[1]
Somewhat less than I used to	26 (11%)	13 (12%)	44 (15%)	588 (20%)	340 (20%)	248 (21%)
Much less than I used to	6 (2.5%)	9 (8.6%)	8 (2.7%)	159 (5.5%)	96 (5.6%)	63 (5.4%)
Rarely	3 (1.2%)	0 (0%)	5 (1.7%)	47 (1.6%)	23 (1.3%)	24 (2.1%)
Unknown	0	0	1	9	2	7
**I have lost interest in my appearance:**						
I take just as much care as ever	187 (77%)	76 (72%)	210 (70%)	1,937 (67%)	1,180 (69%)	757 (65%)
Sometimes, I don't take as much care as I should	44 (18%)	17 (16%)	60 (20%)	548 (19%)	321 (19%)	227 (20%)
Often, I don't take as much care as I should	6 (2.5%)	8 (7.6%)	18 (6.0%)	268 (9.3%)	140 (8.1%)	128 (11%)
Definitely	5 (2.1%)	4 (3.8%)	11 (3.7%)	126 (4.4%)	77 (4.5%)	49 (4.2%)
Missing	0	0	1	10	2	8
**I can sit at ease and feel relaxed:**						
Always	115 (48%)	48 (46%)	133 (44%)	1,206 (42%)	711 (41%)	495 (42%)

Characteristic	*SALT <= 10, N = 242*[1]	*SALT > 10 & <=20, N = 105*[1]	*SALT > 20 & <50, N = 300*[1]	*SALT >= 50 to 100, N = 2,889*[1]	*SALT >= 50 to 99, N = 1,720*[1]	*SALT 100, N = 1,169*[1]
Usually	104 (43%)	46 (44%)	133 (44%)	1,322 (46%)	795 (46%)	527 (45%)
Not often	21 (8.7%)	10 (9.5%)	28 (9.3%)	322 (11%)	187 (11%)	135 (12%)
Never	2 (0.8%)	1 (1.0%)	6 (2.0%)	37 (1.3%)	26 (1.5%)	11 (0.9%)
Missing	0	0	0	2	1	1
**I get a sort of frightened feeling as if something awful is about to happen:**						
Never	164 (68%)	61 (58%)	194 (65%)	1,773 (61%)	1,053 (61%)	720 (62%)
Sometimes, but it doesn't worry me	55 (23%)	33 (31%)	80 (27%)	742 (26%)	454 (26%)	288 (25%)
Yes, but not too badly	19 (7.9%)	9 (8.6%)	23 (7.7%)	306 (11%)	166 (9.7%)	140 (12%)
Very definitely and fairly badly	4 (1.7%)	2 (1.9%)	3 (1.0%)	68 (2.4%)	47 (2.7%)	21 (1.8%)

Characteristic	*SALT <= 10, N = 242*[1]	*SALT > 10 & <=20, N = 105*[1]	*SALT > 20 & <50, N = 300*[1]	*SALT >= 50 to 100, N = 2,889*[1]	*SALT >= 50 to 99, N = 1,720*[1]	*SALT 100, N = 1,169*[1]
**Worrying thoughts go through my mind**						
Almost never	132 (55%)	55 (52%)	155 (52%)	1,312 (45%)	777 (45%)	535 (46%)
Not too often	86 (36%)	34 (32%)	109 (36%)	1,139 (39%)	670 (39%)	469 (40%)
A lot of the time	16 (6.6%)	13 (12%)	30 (10%)	331 (11%)	204 (12%)	127 (11%)
A great deal of the time	8 (3.3%)	3 (2.9%)	6 (2.0%)	107 (3.7%)	69 (4.0%)	38 (3.3%)

[1]n (%)

AA, Alopecia Areata; HADS, Hospital Anxiety & Depression Scale; SALT, Severity of Alopecia Tool
