# Supplementary material for: Estimation of health utility values for alopecia areata
Source: Qual Life Res. 2024 Mar 29;33(6):1581–92. doi: 10.1007/s11136-024-03645-9 (PMC11116246; doi:10.1007/s11136-024-03645-9)
Supplement: Supplementary file 4 — Supplementary file4 (PDF 139 kb) [file 11136_2024_3645_MOESM4_ESM.pdf]

Article title: Estimation of health utility values for alopecia areata

Journal name: Quality of Life Research

Author names: Daniel Aggio, Caleb Dixon, Ernest H. Law, Rowena Randall, Thomas Price, Andrew Lloyd

Corresponding Author: Daniel Aggio (Daniel.Aggio@acasterlloyd.com); Acaster Lloyd Consulting Ltd. 8th Floor, Lacon House, 84 Theobalds Road, London WC1X 8NL

Online Resource 4. Concept elicitation sample demographics

Characteristic		Adult patients (N=3)	Adolescent patients (N=3)	Caregivers (N=5)
Age	Mean (SD)	28 (5.9)	14.3 (0.5)	44.2 (3.5)
	Range	20 - 34	14 - 15	39 - 49
Gender	Male	1 (33.3%)	1 (33.3%)	0 (0%)
	Female	2 (66.6%)	2 (66.6%)	5 (100%)
Scalp Hair Assessment PRO (current hair loss)[1]	No missing hair	0 (0%)	0 (0%)	0 (0%)
	A limited area (1-20%)	0 (0%)	0 (0%)	0 (0%)
	A moderate area (21-49%)	0 (0%)	0 (0%)	0 (0%)
	A large area (50-94%)	2 (66.6%)	1 (33.3%)	2 (40%)
	Nearly all or all (95-100%)	1 (33.3%)	2 (66.6%)	3 (60%)

[1]*Caregiver column denotes level of hair loss experienced by the adolescent they care for*

SD, Standard deviation; PRO, patient reported outcome
