# Supplementary material for: Estimation of health utility values for alopecia areata
Source: Qual Life Res. 2024 Mar 29;33(6):1581–92. doi: 10.1007/s11136-024-03645-9 (PMC11116246; doi:10.1007/s11136-024-03645-9)
Supplement: Supplementary file 6 — Supplementary file6 (PDF 185 kb) [file 11136_2024_3645_MOESM6_ESM.pdf]

Article title: Estimation of health utility values for alopecia areata

Journal name: Quality of Life Research

Author names: Daniel Aggio, Caleb Dixon, Ernest H. Law, Rowena Randall, Thomas Price, Andrew Lloyd

Corresponding Author: Daniel Aggio (Daniel.Aggio@acasterlloyd.com); Acaster Lloyd Consulting Ltd. 8th Floor, Lacon House, 84 Theobalds Road, London WC1X 8NL

Online Resource 6. Final health state vignettes

Background Information: patient vignette

The following health states describe **someone** with a disorder that can cause hair loss, which can affect different aspects of health, including mental and social wellbeing. The descriptions vary in terms of the severity of the symptoms and impacts on quality of life.

Please imagine yourself as the person in the description.

SALT 0-10 Health state

- You have missing hair on your scalp in a single patch or a few small patches (10% or less of your scalp hair is missing). You may be able to hide the hair loss with your other hair. You have normal eyebrows and eyelashes.
- You rarely don't take part in physical activities or exercise because of your hair loss.
- You do not limit your social interactions with friends and family because of your hair loss and you do not find it difficult to meet new people.
- You rarely lack confidence in your appearance because of your hair loss.
- You rarely feel frustrated and angry about your hair loss.
- You rarely feel self-conscious or embarrassed about your hair loss.
- You rarely feel sad and depressed about your hair loss and you are almost always able to enjoy yourself.
- You occasionally feel anxious and stressed. Worrying thoughts occasionally go through your mind. Your sleep is very rarely disturbed by these worrying thoughts, and you very rarely feel tired.
- You rarely do not feel like yourself.
- You rarely experience mild discomfort on your skin or nails.

SALT 11-20 Health state

- You have patches of missing hair on your scalp (between 11-20% of your scalp hair is missing). You have normal eyebrows and eyelashes.
- Occasionally you do not take part in physical activities or exercise because of your hair loss.
- You limit your social interactions with friends and family because of your hair loss some of the time and you occasionally find it difficult to meet new people.
- You sometimes lack confidence in your appearance because of your hair loss.
- You rarely feel frustrated and angry about your hair loss.
- You occasionally feel self-conscious or embarrassed about your hair loss.
- You occasionally feel sad and depressed about your hair loss but you are frequently able to enjoy yourself.
- You sometimes feel anxious and stressed. Worrying thoughts sometimes go through your mind. Your sleep is rarely disturbed by these worrying thoughts, and you rarely feel tired.
- You occasionally do not feel like yourself.
- You rarely experience mild discomfort on your skin or nails.

SALT 21-49 Health state

- You have considerable hair loss on your scalp (between 21-49% of your scalp hair is missing). You have normal eyebrows and eyelashes.
- Sometimes you do not take part in physical activities or exercise because of your hair loss.
- You limit your social interactions with friends and family because of your hair loss quite a lot and you often find it difficult to meet new people.
- You often lack confidence in your appearance because of your hair loss.
- You often feel frustrated and angry about your hair loss.
- You often feel self-conscious or embarrassed about your hair loss.
- You often feel sad and depressed about your hair loss. Sometimes the sadness and depression are severe. You are rarely able to enjoy yourself because of your hair loss.
- You often feel anxious and stressed. Worrying thoughts often go through your mind. Your sleep is occasionally disturbed by these worrying thoughts, and you occasionally feel tired.
- You go through periods of not feeling like yourself.
- You occasionally experience mild discomfort on your skin or nails.

SALT 50-100 Health state

- You have extensive hair loss on your scalp (between 50% to 100% of your scalp hair is missing). You have normal eyebrows and eyelashes.
- Most of the time you do not take part in physical activities or exercise because of your hair loss.
- You limit your social interactions with family, friends and colleagues because of your hair loss a lot and you often find it difficult to meet new people.
- You frequently lack confidence in your appearance because of your hair loss.
- You frequently feel frustrated and angry about your hair loss.
- You frequently feel self-conscious and embarrassed about your hair loss.
- You often feel sad and depressed about your hair loss. Sometimes the sadness and depression are severe. You are rarely able to enjoy yourself because of your hair loss.
- You frequently feel anxious and stressed. Worrying thoughts frequently go through your mind. Your sleep is sometimes disturbed by these worrying thoughts, and you sometimes feel tired.
- You go through periods of not feeling like yourself.
- You sometimes experience mild discomfort on your skin or nails.

SALT 50-100 Health state + eyebrow & eyelash hair loss

- You have extensive hair loss on your scalp (between 50% to 100% of your scalp hair is missing). Most or all of your eyebrow and eyelash hair is also missing.
- Most of the time you do not take part in physical activities or exercise because of your hair loss.
- You limit your social interactions with friends, family and colleagues because of your hair loss a lot and you frequently find it difficult to meet new people.
- You very frequently lack confidence in your appearance because of your hair loss.
- You very frequently feel frustrated and angry about your hair loss.
- You very frequently feel self-conscious or embarrassed about your hair loss.
- You frequently feel sad and depressed about your hair loss. Sometimes the sadness and depression are severe. You are rarely able to enjoy yourself because of your hair loss.
- You very frequently feel anxious and stressed. Worrying thoughts very frequently go through your mind. Your sleep is sometimes disturbed by these worrying thoughts, and you sometimes feel tired.
- You go through periods of not feeling like yourself.
- You sometimes experience mild discomfort on your skin, nails or around your eyes.

Introduction: caregiver vignette

The following health state describes **someone who cares for a family member** with a disease that causes hair loss. The family member is aged between 12 to 17 years old and has extensive hair loss on their scalp (between 50% to 100% of their scalp hair is missing). They may have some loss of eyebrow and eyelash hair. We would like you to imagine that this is your child and you look after them.

Please imagine yourself as the parent or caregiver in the description

Caregiver for adolescent (aged 12-17 years) with ≥50 SALT, no eyebrow or eyelash hair loss

- You need to spend some time on most days supporting your family member (e.g., helping to conceal hair loss, providing emotional or social support)
- You occasionally have less time for other family members
- Most of the time you are able to do your usual activities, such as going to work, socialising and leisure activities, but you are sometimes unable to do certain activities (e.g., swimming and other outdoor activities) with your family because of your family member's condition
- You do not have pain or discomfort, problems walking around or problems with self-care
- You frequently feel stressed and worried about your family member's condition
- You occasionally feel frustrated about your family member's condition
- You sometimes feel sad
- Your sleep is sometimes disrupted, and you sometimes feel tired
