# Supplementary material for: Estimation of health utility values for alopecia areata
Source: Qual Life Res. 2024 Mar 29;33(6):1581–92. doi: 10.1007/s11136-024-03645-9 (PMC11116246; doi:10.1007/s11136-024-03645-9)
Supplement: Supplementary file 7 — Supplementary file7 (PDF 145 kb) [file 11136_2024_3645_MOESM7_ESM.pdf]

Article title: Estimation of health utility values for alopecia areata

Journal name: Quality of Life Research

Author names: Daniel Aggio, Caleb Dixon, Ernest H. Law, Rowena Randall, Thomas Price, Andrew Lloyd

Corresponding Author: Daniel Aggio (Daniel.Aggio@acasterlloyd.com); Acaster Lloyd Consulting Ltd. 8th Floor, Lacon House, 84 Theobalds Road, London WC1X 8NL

Online Resource 7. Time trade off utility weights sensitivity analysis

Health State	Mean (SD)	Range	95% CI
N = 114			
SALT 0-10	0.918 (0.121)	0.175 - 1.000	0.896 - 0.940
SALT 11-20	0.847 (0.231)	0.125 - 0.975	0.804 - 0.890
SALT 21-49	0.689 (0.314)	-1.000 - 0.975	0.632 - 0.747
SALT 50-100	0.533 (0.470)	-1.000 - 0.975	0.447 - 0.619
SALT 50-100 + eyebrow & eyelash loss	0.481 (0.471)	-1.000 - 0.975	0.394 - 0.568
N = 57[1]			
Caregiver	0.882 (0.128)	0.375 - 1.000	0.849 - 0.915

[1] The caregiver vignette was finalised and introduced into valuation interviews after fieldwork with patient health states was initiated resulting in a reduced sample size

SALT, Severity of Alopecia Tool; SD, Standard Deviation
